# Optimizing sterile filtration of nanoemulsions through proper choice of prefilter properties

**DOI:** 10.1002/btpr.70087

**Published:** 2025-10-31

**Authors:** Shreya Kapila, Randal J. Soukup, Marissa E. Bradley, David Boyd, Andrew L. Zydney

**Affiliations:** ^1^ Department of Chemical Engineering The Pennsylvania State University University Park Pennsylvania USA; ^2^ Vaccine Process Research and Development Merck & Co., Inc. Rahway New Jersey USA

**Keywords:** bioprocessing, drug delivery, membrane fouling, nanoemulsions, prefiltration, sterile filtration

## Abstract

Nanoemulsions, with droplet sizes between 20 and 200 nm, have emerged as a promising vaccine adjuvant and drug delivery system, enhancing the solubility of hydrophobic drugs for diverse applications. Sterile filtration of nanoemulsions is particularly challenging due to the similar size between the nanodroplets and the 0.2 μm nominal pore size rating of sterile filters. One approach to reducing membrane fouling, and enhancing filtration capacity and yield, is to employ an appropriate prefilter, but there are currently no clear guidelines on how to select the prefilter pore size, chemistry, or morphology for sterile filtration of nanoemulsions. This study examined the performance of a range of prefilters with varying pore morphologies and surface chemistries. Sessile drop contact angles were used to evaluate the prefilter hydrophobicity, and bubble point and mercury intrusion porosimetry were used to evaluate the pore characteristics of the different prefilters. The best performance was achieved using a relatively hydrophobic 0.45 μm prefilter made of polyvinylidene fluoride but modified with a somewhat hydrophilic (oxygen‐containing) coating. This prefilter reduced the surface tension of the nanoemulsion and provided more than a 2‐fold increase in capacity for a variety of sterile filters. These results provide critical insights into the factors influencing nanoemulsion filtration and offer a framework for selection of appropriate prefilters in biopharmaceutical manufacturing.

## 1 INTRODUCTION

Nanoemulsions (NEs) are stable dispersions formed by mixing oil and water phases with surfactants to form a kinetically stabilized emulsion with droplets <1 μm in size. As a drug delivery system, NEs can encapsulate fat‐soluble drugs, improving their solubility and enhancing oral or transdermal absorption.^1^, ^2^, ^3^, ^4^, ^5^ They can also encapsulate water‐soluble macromolecular protein drugs to improve bioavailability.^6^, ^7^ Several intravenous NE formulations, such as Intralipid, Diazemuls, Cleviprex, and Diprivan, have been approved for pharmaceutical use.^8^, ^9^ NEs are also employed as vaccine adjuvants to boost the immune response, especially in individuals less responsive to aluminum adjuvants, like children and the elderly.^10^, ^11^, ^12^

Sterility is an essential requirement for all parenteral NEs to ensure their safe administration.^13^, ^14^, ^15^ Heat sterilization and gamma radiation cannot be used for NE sterilization as they degrade the product, while aseptic processing is highly expensive, requires more complex facility design and operation with highly trained personnel, and it can be challenging to assure sterility throughout the process. In contrast, filtration through a sterilizing‐grade filter can not only remove all microorganisms; it can also reduce the number of large nanodroplets and enhance the stability of NEs. However, several recent studies have shown that NEs cause rapid fouling of sterile filters, leading to low capacities and higher costs. Kapila et al.^16^ demonstrated that the filtrate flux was essentially zero until the transmembrane pressure surpassed a critical threshold, corresponding to the force necessary to drive the deformable nanoemulsion droplets through the membrane pores. Although all sterile filters are rated as 0.2 μm pore size, the different sterile filters had different threshold pressures, consistent with differences in the maximum pore size determined from bubble point measurements.^16^, ^17^ Degenève^18^ prepared squalene NEs using classical surfactants (Tween 80 and Span 85) and innovative PVA surfactants (PVA 4–88 and PVA 4–98) in various combinations and ratios. The capacity determined with 0.2 μm cellulose acetate membranes varied dramatically based on the formulation, with values as low as 0.30 L/m^2^ for the PVA surfactants to as much as 240 L/m^2^ with the classical surfactants.

Su et al.^19^ examined the sterile filtration of a squalane‐based NE, with significant improvements in performance when using the Fluorodyne® EX EDF membrane as a prefilter. Similar benefits of prefiltration have been reported by Kapila et al.^16^ for NEs and by Taylor et al.^20^ for a live‐attenuated viral vaccine, with dual‐layer sterile filters showing better capacity and yield compared to single‐layer filters. Du et al.^21^ demonstrated that prefiltration with 5 μm pore size Durapore® membranes significantly reduced fouling and improved the capacity during sterile filtration of a glycoconjugate vaccine due to the removal of large foulants greater than 1 μm in size. Cutler et al.^22^ showed that 0.45 μm pore size prefilters could significantly reduce fouling during the sterile filtration of various model proteins through the removal of large protein aggregates.

The objective of this study was to evaluate the impact of various prefilters on the sterile filtration of a NE and to determine the critical properties of the prefilter for enhancing sterile filter performance in this application. Prefilters with varying pore sizes, morphologies, and surface chemistries were tested, with dynamic light scattering (DLS) used to assess any changes in the NE droplet size distribution. The pore size characteristics of the prefilters were evaluated using bubble point tests and mercury intrusion porosimetry, with the surface chemistry examined using contact angle measurements and X‐ray photoelectron spectroscopy (XPS). The findings clearly highlight the potential of using prefilters with appropriate chemistry and pore size to significantly reduce fouling and increase capacity during sterile filtration of NEs.

## 2 MATERIALS AND METHODS

### 2.1 Nanoemulsions

Oil‐in‐water NEs were prepared as described by Kapila et al.^16^ First, squalene, Tween 20 (polyoxyethylene sorbitan monolaurate), and Span 85 (sorbitan trioleate) were mixed with 20 mM L‐histidine buffer at pH 5.8 (all obtained from MilliporeSigma, St. Louis, MO) in a Silverson mixer (L5MA Silverson Machines, Inc., East Longmeadow, MA) to form a coarse emulsion with a mean droplet diameter of 1100 ± 100 nm as measured by dynamic light scattering (DLS). In addition to the primary peak, minor peaks were also observed around 90 and 340 nm. The coarse emulsion was then processed through a GEA Panda Plus 2000 high‐pressure homogenizer (Parma, Italy) for 10 discrete passes at 140,000 kPa (20,000 psi) to obtain a NE with a mean droplet size around 160 nm. The NEs were stored at 4°C and then warmed to room temperature (21°C ± 2°C) for use in the filtration experiments. NE concentrations were evaluated by UV absorbance at 450 nm based on the squalene and surfactant concentration using a Tecan microplate reader (Mannedorf, Switzerland) with 96‐well clear microplates.

The size distribution of the NE was evaluated by Dynamic Light Scattering (DLS) using a Malvern Zetasizer Nano ZS90 (Malvern, UK). Samples were diluted by 25× to obtain more accurate scattering data. The surface tension of the NE was evaluated using an Automated Goniometer (Rame‐Hart, model 295‐F4). The instrument was calibrated with a 4 mm diameter combo calibration sphere (p/n 100‐27‐31‐U), achieving an aspect ratio of 0.9999 after calibration. Measurements were obtained using DROPimage Advance (v2.6.1) software, analyzing three consecutive drops with 10 measurements taken at 1‐s intervals. A 22‐gage stainless steel syringe was used for drop formation. The reported surface tension values represent the average ± standard deviation of these measurements.

### 2.2 Normal flow filtration

Sterile filtration was performed using the Supor 0.2 μm polyethersulfone sterile filter (purchased from Pall, now Cytiva), along with several other commercial sterile filters as summarized in Table 1. Data were obtained both with and without different prefilters having different pore sizes and surface chemistries (upper section of Table 1). The term Durapore was used throughout the text to refer to the hydrophilic variant unless explicitly stated otherwise. Note that Sterlitech recently changed their PVDF prefilter; the new version exhibits substantially different performance than that reported in this study. Prefilters were housed in a stainless‐steel holder sealed with an O‐ring. All filters were flushed with the 20 mM L‐histidine buffer using a minimum of 100 L/m^2^. Some experiments were performed with the commercial Supor 0.8 / 0.2 μm dual‐layer filter in a pre‐sealed syringe format.

**TABLE 1:** Summary of different prefilters (top panel) and sterile filters (bottom panel) examined in this work.

Prefilters	Pore size (μm)	Chemistry	Part number
Durapore	0.45	PVDF (Hydrophilic)	HVLP02500
	0.45	PVDF (Hydrophobic)	HVHP02500
Sterlitech	0.45	PVDF	PVDF4547100
	0.45	Nylon	NY4525100
	0.45	PTFE	PTFE04525100
Pall Supor	0.8	PES	60,109

Sterile filters	Pore size (μm)	Chemistry	Part number
Pall Supor	0.2	PES	60,300
Pall Supor Syringe	0.8|0.2	PES	4905‐N
Sartopore 2 XLG	0.8|0.2	PES	5445307GV—LX‐C
Supor EKV	0.65|0.2	PES	FTKEKV
Sartobran P	0.45|0.2	Cellulose Acetate	5235307HV—LX—C
Millipore Express Plus	0.2	PES	GPWP04700
Durapore	0.2	PVDF	GVWP02500

Filtration experiments were performed at a constant pressure of 280 kPa (40 psi) based on our previous work^16^; operation below the threshold pressure (typically around 100 kPa) gave no measurable filtrate flux. The applied pressure was set by air pressurization of the feed reservoir, with the pressure monitored using an Ashcroft digital pressure gage (Stratford, CT). The filtrate flow rate was evaluated by timed mass collection using an OHAUS Ranger™ 3000 scale (Parsippany, NJ) with data logging through the OHAUS Serial Port Data Collection Software.

### 2.3 Filter characterization

The water contact angles for the different prefilters were measured using a Ramé‐Hart Model 260 automated goniometer (Succasunna, NJ). Water droplets of 10 μL volume were carefully dropped onto the dry membrane surface. The droplet size, contact angle, and stage tilt were evaluated within 0.5 s and analyzed using DROPimage software. Three replicate measurements were obtained for each sample, with results reported as the mean plus/minus the standard deviation. Dynamic contact angle measurements were performed similarly, with the contact angle measured for individual droplets over a period of 180 s.

Mercury intrusion porosimetry (MIP) was used to evaluate the pore size distribution of the different prefilters. MIP was conducted using a two‐stage Micromeritics AutoPore V Model 9620 (Norcross, GA). The disc filters were cut and folded, with 0.2–0.3 g of the filter media placed into the bulb of the penetrometer. The mercury pressure was increased incrementally from 2 to 20 kPa, allowing the sample to equilibrate with the intruded mercury at each pressure. The incremental intrusion volume was plotted as a function of the intrusion pressure, with the pore size distribution evaluated by differentiation of the cumulative volume data. The mercury was collected by vacuum for reuse in subsequent experiments, with the used filters discarded as an environmental hazard.

The maximum pore size for each filter was evaluated from bubble point measurements obtained using 25 mm discs in a stainless‐steel holder connected to a compressed nitrogen tank. The nitrogen pressure was gradually increased at a rate of approximately 7 kPa per minute using a ProStar platinum pressure regulator. A dip tube was attached to the outlet from the stainless‐steel holder, with the bubble point defined as the pressure at which nitrogen bubbles were first observed in the 150 mL beaker of deionized (DI) water. The maximum pore diameter d_bp_ was calculated using the Young‐Laplace equation:

(1) $$d_{\mathrm{bp}}=\frac{4\;\gamma \;\cos\;\theta }{\mathrm{\Delta P}}$$

where ΔP is the pressure difference at which bubbles first appear, γ = 72 mN/m is the water surface tension, and θ is the contact angle (which was assumed to be zero for all membranes).

## 3 RESULTS AND DISCUSSION

### 3.1 Role of prefilters

Our recent study of the sterile filtration of a squalene‐in‐water NE showed capacities ranging from <50 g/m^2^ for the Millipore Express Plus to as much as 1200 g/m^2^ for the Pall/Cytiva Supor and 2500 g/m^2^ for the Sartorius Sartopore 2 XLG. In each case, the capacity was defined as the mass of NE filtered per unit membrane area at 90% flux decline, with the mass calculated as the product of the volume filtered and the concentration of squalene + surfactant as determined from the absorbance at 450 nm. All three sterile filters were polyethersulfone membranes. The Supor and Sartopore 2 XLG were both dual‐layer filters with a 0.8 μm pore size prefilter on top of a 0.2 μm pore size‐rated sterilizing‐grade membrane, while the Millipore Express Plus was a highly asymmetric single‐layer filter. The much greater capacity of the two dual‐layer filters suggests that the prefilter has a significant affect on the overall filtration performance.

**FIGURE 1:**
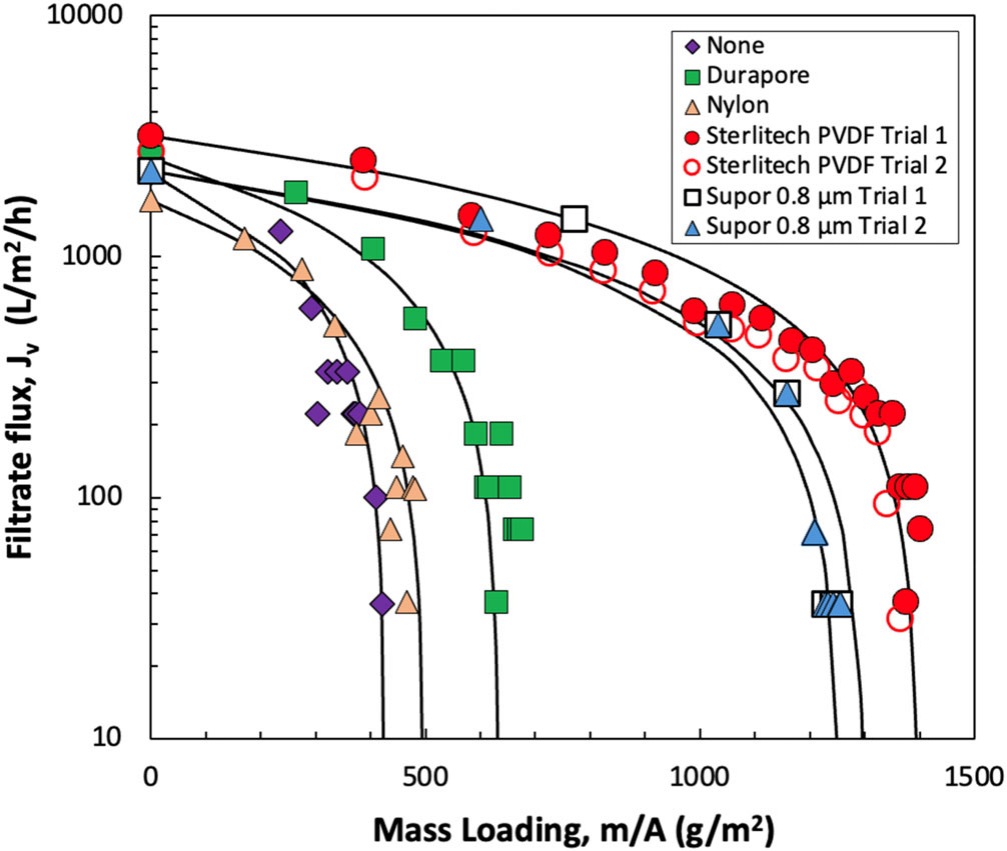
Filtrate flux as a function of mass loading for sterile filtration of nanoemulsions through the Supor 0.2 μm PES membrane (without any prefilter) and after prefiltration through the different prefilters shown in Table 1. Prefiltration was performed in batch mode at a constant pressure of 280 kPa (the same pressure used for the sterile filtration). Replicates are shown for the Sterlitech 0.45 μm and Supor 0.8 μm prefilters. Solid curves are model fits generated using the complete pore blockage model (Equation 2) with best fit parameters shown in Table 2.

In order to understand the impact of the prefilter layer in more detail, a series of experiments were performed in which the NE was first passed through the prefilter (at a constant pressure of 280 kPa), with the permeate collected and then filtered through the Supor 0.2 μm sterile filtration membrane (without any prefilter) under the same conditions. The filtrate flux through the prefilters was all greater than 11,000 L/m^2^/h, with less than a 12% decline over mass loadings of 5000 g/m^2^. Figure 1 shows filtrate flux data for the different pre‐filtered NEs during constant pressure filtration through separate Supor 0.2 μm sterilizing grade membranes. Results for replicate experiments performed after prefiltration through the Supor 0.8 μm prefilter and the Sterlitech 0.45 μm PVDF prefilter are in good agreement. Replicates performed with the other prefilters showed similar reproducibility but are not shown to improve the clarity of the figure.

The solid curves in Figure 1 are model fits developed using the complete pore blockage model as discussed by Kapila et al.^16^

(2) $$J=J_{0}\left(1-\frac{k_{p}m}{A}\;\right)$$

where *J*_o_ is the initial flux (determined by extrapolation of the data back to zero throughput), *k*_p_ is the pore blockage parameter determined by minimizing the sum of the squared residuals between the model and data, and m is the mass of the filtered product. The best fit values of *J*_0_ and *k*_p_ are shown in Table 2. The filter capacity was calculated as 1/*k*_p_, which is the value of the mass loading at which the filtrate flux goes to zero. We did not evaluate the capacity using the classical Vmax analysis, although such plots provided fairly similar estimates of the capacity (but the fits to the fouling data were much poorer than those shown in Figure 1). The lowest capacity was obtained when using the Supor 0.2 μm sterile filter alone. The batch prefiltration through the 0.45 μm Nylon prefilter provided only a small increase in the capacity (<20%). In contrast, the 0.45 μm Sterlitech prefilter and the 0.8 μm Supor prefilter both provided more than a 3‐fold increase in capacity. In addition, the initial flux through the sterile filter after prefiltration through the 0.45 μm Sterlitech prefilter increased by almost a factor of two compared to that obtained using the 0.45 μm Nylon prefilter.

**TABLE 2:** Best fit values of the initial flux (*J*_0_) and pore blockage parameter (*k*_p_) during filtration through Supor 0.2 μm sterile filters after different prefilters (model curves in Figure 1).

Prefilter	*k*_p_ (m^2^/g)	*J*_0_ (L/m^2^/h)
None	0.0023	2200
Durapore	0.0016	2600
Nylon	0.0020	1700
Sterlitech PVDF	0.00072	3200
Supor 0.8 μm	0.00080	2300

To understand the large differences in effectiveness of the different prefilters, the NE particle size distributions before and after prefiltration were evaluated using dynamic light scattering with the Z‐average diameter for the NE after prefiltration through the Supor 0.8 μm, Sterlitech PVDF 0.45 μm, Nylon 0.45 μm, and Durapore 0.45 μm shown in Table 3; the Z‐average diameter for the freshly prepared NE was 166 ± 4 nm. The Z‐average diameters were all very similar, with the Z‐average diameter for the NE after prefiltration being slightly larger than that for the fresh NE for most of the prefilters. This is likely due to the removal / fusion of some of the smaller nanodroplets. The Z‐average diameter for the NE obtained after filtration through the Supor 0.8 μm prefilter was statistically identical to that of the fresh NE. The details of the nanodroplet size distributions were also similar for all conditions, with no statistically significant differences between the permeate solutions collected through the different prefilters.

**TABLE 3:** Z‐average size of the NE after filtration through the different prefilters determined by dynamic light scattering.

Prefilter	Z‐average Diameter (nm)
Durapore 0.45 μm	174 ± 3
Sterlitech PVDF 0.45 μm	173 ± 3
Nylon 0.45 μm	168 ± 3
Pall Supor 0.8 μm	165 ± 3

### 3.2 Characterization of prefilter pore size distribution

The very different behavior of the three prefilters with a pore size rating of 0.45 μm (data in Figure 1 and Table 3) was explored further by evaluating the pore size characteristics of the prefilters using two complementary techniques: mercury intrusion porosimetry,^17^ which provides information on the entire pore size distribution, and bubble point measurements, which measure the largest pore size. The average pore diameters determined using mercury intrusion porosimetry, d_Hg_, and the bubble point values, both the measured pressure and the calculated bubble point diameter, d_bp_, are summarized in Table 4.

**TABLE 4:** Pore size characteristics of the different prefilters determined by bubble point and mercury intrusion porosimetry measurements.

Prefilter	Bubble point (kPa)	Bubble point pore diameter, d_bp_(μm)	Volume average pore diameter, d_Hg_ (μm)
Durapore 0.45 μm	176 ± 2	1.64 ± 0.01	0.44
Sterlitech PVDF 0.45 μm	173 ± 13	1.66 ± 0.12	0.85
Nylon 0.45 μm	214 ± 25	1.35 ± 0.17	0.85
Pall Supor 0.8 μm	110 ± 1	2.62 ± 0.02	1.16

The Pall Supor 0.8 μm prefilter had the smallest bubble point pressure (largest value of d_bp_) as expected. The 0.45 μm Nylon membrane shows the highest bubble point, and thus the smallest maximum pore size, even though this prefilter had minimal impact on the capacity of the Supor 0.2 μm sterile filter (data in Figure 1). The 0.45 μm Durapore and Sterlitech prefilters, which had very different effects on the sterile filter capacity, show very similar values of d_bp_, demonstrating that the maximum pore size has no observable correlation with the prefilter performance. The much larger calculated pore size (based on the bubble point) compared to the manufacturer's pore size rating is typical of porous membranes.^23^

**FIGURE 2:**
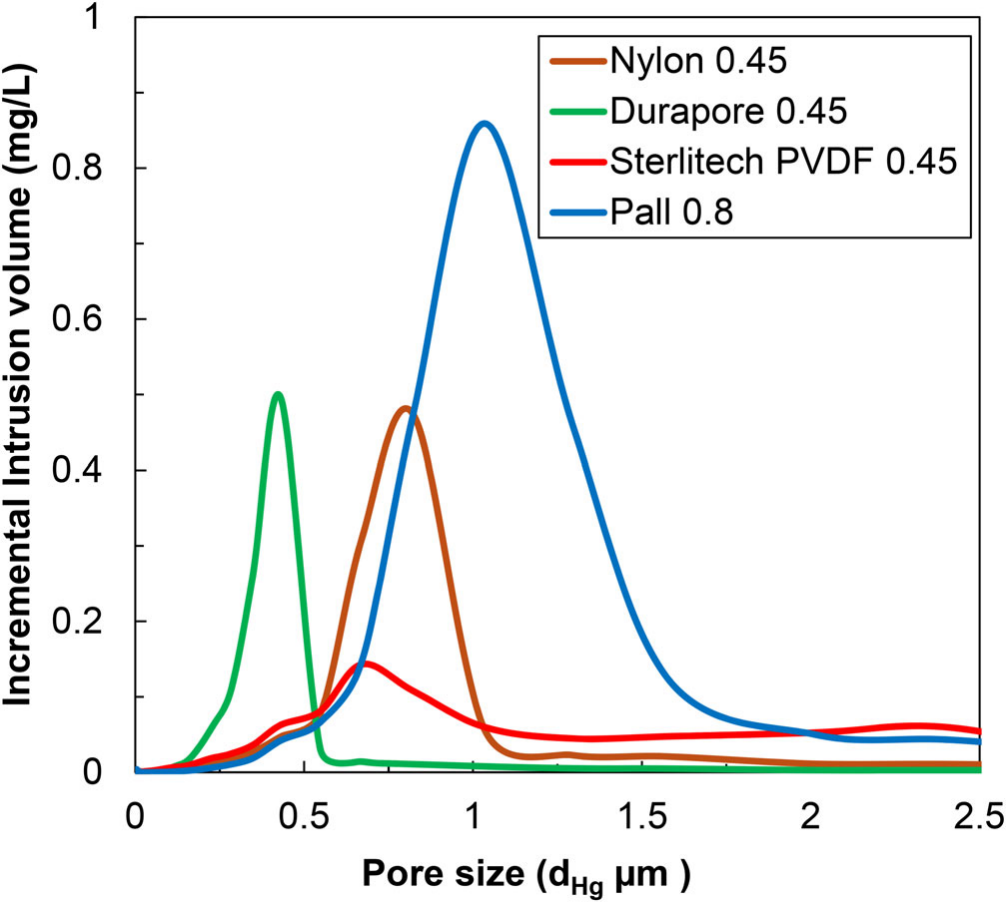
Pore size distributions for the different prefilters as determined by mercury intrusion porosimetry.

Figure 2 compares the pore size distributions for the different prefilters based on mercury intrusion porosimetry data. The 0.45 μm Durapore prefilter shows a monodisperse pore size distribution with a volume average pore diameter of d_Hg_ = 0.44 μm and a most probable pore size of 0.45 μm, both in excellent agreement with the pore size rating. The 0.45 μm Nylon prefilter also shows a monodisperse pore size distribution but with a peak at 0.8 μm and volume average pore diameter of 0.85 μm. In contrast, the 0.45 μm Sterlitech PVDF prefilter has a fairly broad pore size distribution, with a small peak at approximately 0.45 μm, a larger peak at 0.67 μm, and a very broad peak with diameter well above 1 μm, with d_Hg_ = 0.85 μm. This broad pore size distribution is due to the presence of a small number of large voids in this prefilter as shown in the SEM images in the Supplementary Information. The Supor 0.8 μm prefilter has the largest pore size as expected with d_Hg_ = 1.16 μm, even though this prefilter is highly effective in reducing the fouling of the Supor 0.2 μm sterilizing grade membrane. SEM images of the upper and lower surfaces of the Supor 0.8 μm and Sterlitech 0.45 μm prefilters are provided in the Supplementary Information. Note that even though the 0.45 μm pore size Nylon and Sterlitech prefilters show similar values of d_Hg_, they have very different impacts on the capacity of the sterile filter (Figure 1), suggesting that other properties of the prefilters may also play a significant role in determining the filtration behavior.

### 3.3 Prefilter surface chemistry

The elemental composition of the different prefilters was determined using X‐ray Photoelectron Spectroscopy (XPS), with the results summarized in Table 5. Although the 0.45 μm Durapore and Sterlitech prefilters are both listed by their manufacturers as being made of polyvinylidene fluoride (PVDF), there are significant differences in their elemental composition. The Durapore prefilter is 12% oxygen, likely due to a hydroxyalkyl acrylate coating that renders the surface more hydrophilic.^24^ The Sterlitech prefilter also shows an oxygen peak, although the total oxygen content of this prefilter is only 3.7%, less than 1/3 that for the Durapore. Note that the oxygen species present in the Durapore and Sterlitech prefilters may also be different, with the hydroxyalkyl acrylate having both internal ester groups and free hydroxyls. The Nylon prefilter is 12% oxygen and 12% nitrogen, consistent with the amide linkages, while the Supor 0.8 μm prefilter is 16% oxygen, 3.1% nitrogen, and 5.5% sulfur, which is consistent with the chemical composition of polyethersulfone (with the nitrogen likely associated with polyvinylpyrrolidone which is commonly used as a pore forming / wetting agent).

**TABLE 5:** Elemental composition of the different prefilters determined by X‐ray Photoelectron Spectroscopy (XPS).

Sample	C	F	N	O	S
Durapore 0.45 μm	53	35	—	12	—
Sterlitech 0.45 μm	52	44	—	3.7	—
Nylon 0.45 μm	77	—	12	12	—
Supor 0.8 μm	76	—	3.1	16	5.5

**FIGURE 3:**
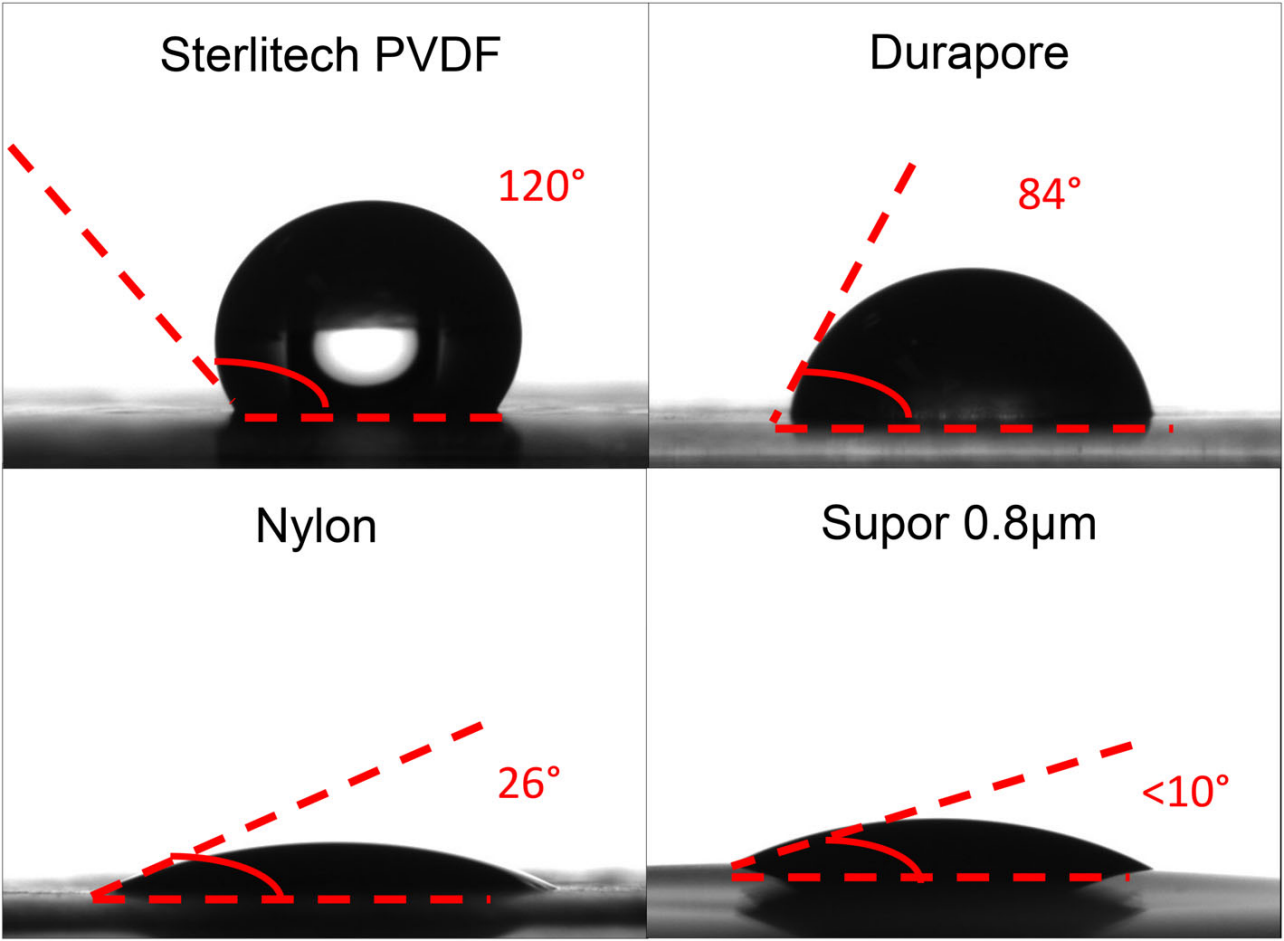
Water contact angles measured 10 ms after depositing a water droplet on the different prefilters.

The impact of these differences in chemical composition on the surface properties of the prefilters was examined using water contact angle measurements. Typical images of the water droplet approximately 10 ms after placement on the surface of the prefilter are shown in Figure 3. The Supor 0.8 μm prefilter showed the smallest contact angle. In order to confirm that this was not simply related to the large pore size of this prefilter (causing the water droplet to rapidly enter the membrane pores), the contact angle was also evaluated for the Supor 0.2 μm sterile filter, giving a value of ≈15°, very similar to that for the Supor 0.8 μm prefilter. The 0.45 μm Sterlitech prefilter has a contact angle of 120°, indicating a significantly greater hydrophobicity than the 0.45 μm Durapore prefilter (contact angle of 84°), which is consistent with the much lower oxygen content of the Sterlitech prefilter. The Nylon prefilter is much more hydrophilic than either of the 0.45 μm PVDF prefilters, with a contact angle of only 26°, reflecting the strong hydrogen bonding character of the amide groups. These differences in hydrophobicity align well with the observed differences in performance of these 0.45 μm prefilters, with the hydrophobic Sterlitech prefilter providing the greatest increase in sterile filter capacity while the hydrophilic Nylon prefilter provided minimal improvement in capacity.

**FIGURE 4:**
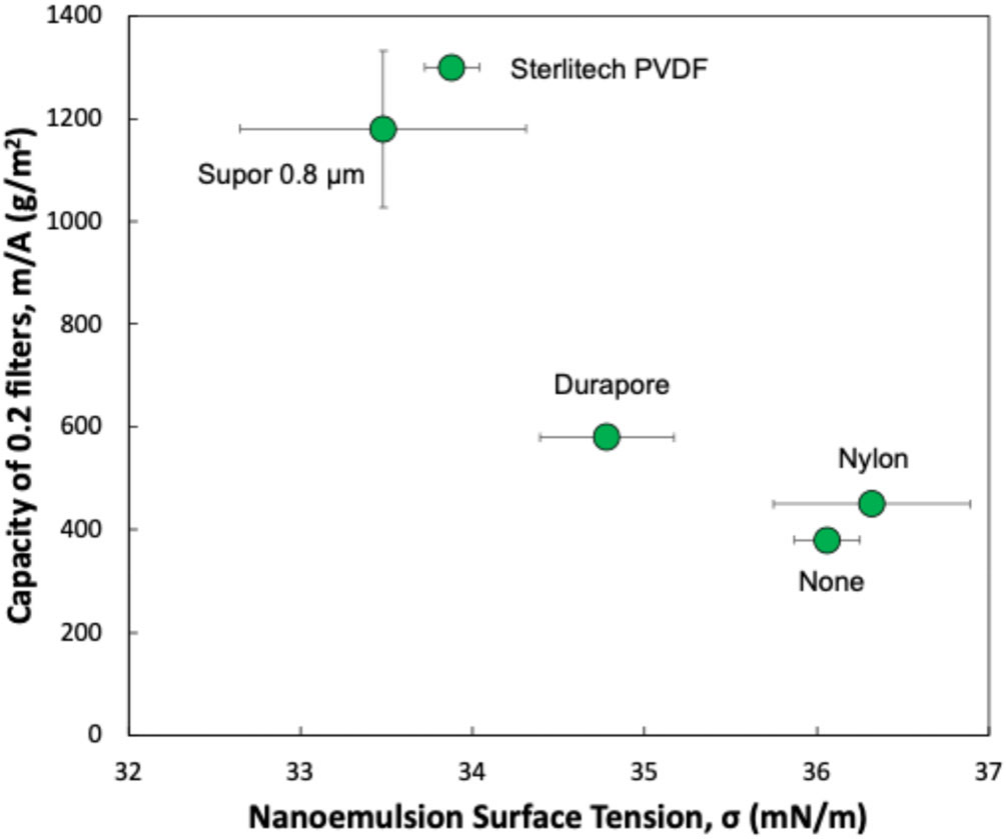
Capacity of the Supor 0.2 μm sterile filter as a function of the surface tension of the nanoemulsion after prefiltration through the different prefilters (labeled by the symbols). Error bars represent standard deviations in repeat measurements; the error bars for the capacity are smaller than the size of the symbols for most of the prefilters.

The correlation between prefilter performance and hydrophobicity (as determined by the contact angle) suggests that the prefilter may alter the properties of the NE by selective removal of specific components due to adsorptive interactions. This was examined further by measuring the surface tension of the NE both before and after prefiltration. Experimental results for the 0.45 μm Durapore, Nylon, and Sterlitech PVDF prefilters along with the Supor 0.8 μm prefilter are summarized in Figure 4, which shows the capacity of the Supor 0.2 μm sterile filter (data from Figure 1) as a function of the measured surface tension of the nanoemulsion in the feed (after prefiltration). The fresh NE had a surface tension of 36.1 ± 0.1 mN/m, which is approximately half that of water (70 mN/m), likely due to the high concentration of surfactants. Prefiltration of the NE through the Supor 0.8 μm prefilter caused a small reduction in surface tension to a value of 33.5 ± 0.2 mN/m, likely due to the removal of more hydrophobic components, for example, free Span 85 which has a hydrophilic–lipophilic balance (HLB) of 1.8 compared to 16.7 for the much more hydrophilic Tween 20. The 0.45 μm Sterlitech PVDF prefilter also caused a small reduction in surface tension (by 6%), while the 0.45 μm Nylon prefilter had no significant effect on the surface tension of the NE.

As seen in Figure 4, the capacity values appear well correlated with the NE surface tension, suggesting that the impact of the prefilter may be primarily through an alteration in the physical composition of the NE. Our hypothesis is that the reduction in surface tension is due to the adsorption / removal of trace quantities of either free squalene or Span 85 during prefiltration; the measured interfacial surface tension of the NE appears to provide a simple measure of these small changes in the NE chemistry. It is certainly possible that the changes in NE surface tension are also influenced by the broad pore size distribution in some of the prefilters, although the data obtained in this work are insufficient to demonstrate this directly. Future studies will be needed to more fully elucidate the underlying mechanisms controlling the NE surface tension and its potential impact on the NE stability.

### 3.4 Effectiveness of the Sterlitech 0.45 μm PVDF prefilter

**FIGURE 5:**
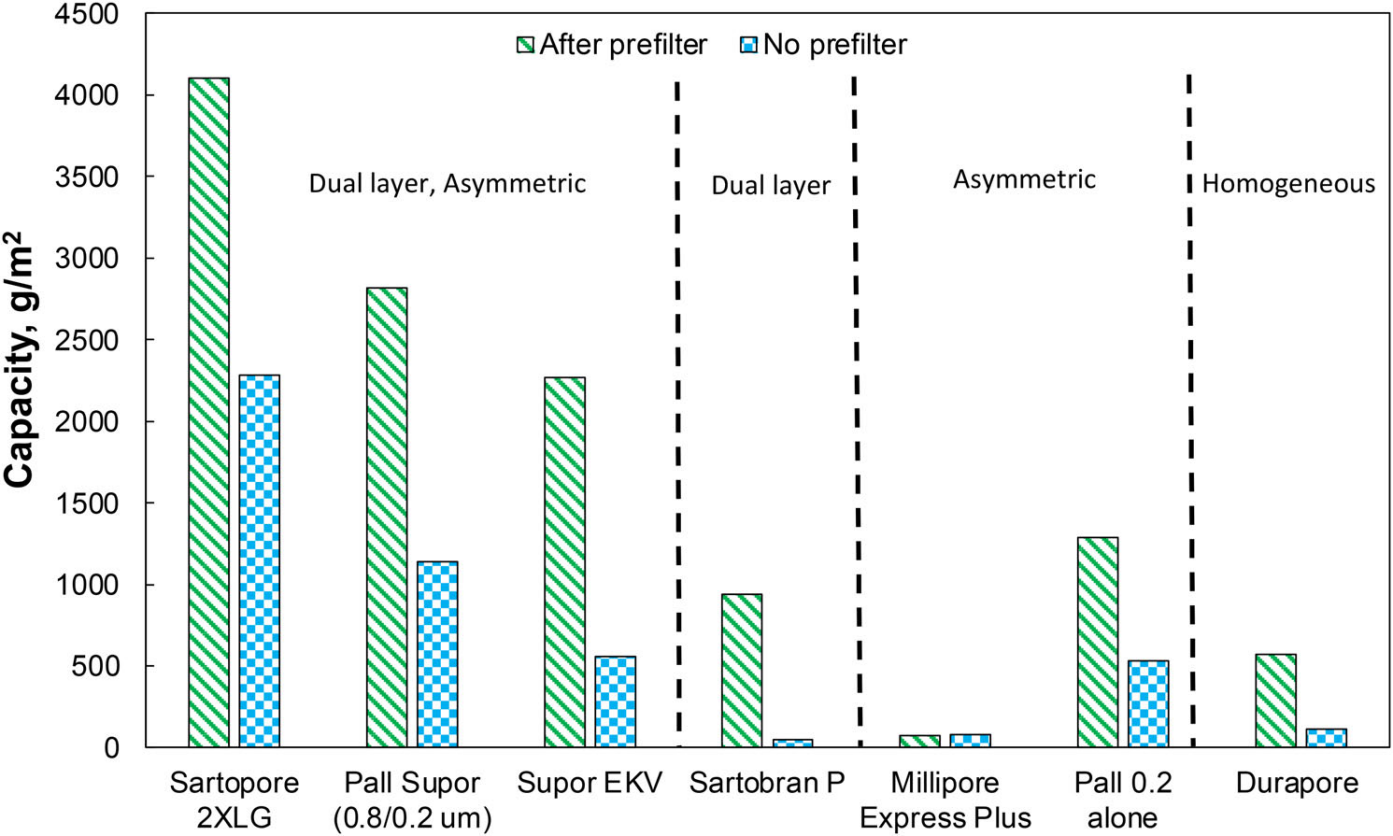
Capacity for the different sterilizing grade filters with both the fresh nanoemulsion and the nanoemulsion after prefiltration through the Sterlitech 0.45 μm PVDF prefilter.

Given the very strong performance of the Sterlitech 0.45 μm PVDF prefilter in improving the performance of the Supor 0.2 μm sterile filter, a series of experiments were performed in which several different sterile filters were challenged with either a fresh NE or a NE that had first been prefiltered through the Sterlitech 0.45 μm PVDF prefilter, with results summarized in Figure 5. The Sterlitech 0.45 μm PVDF prefilter provided a significant increase in capacity for all the sterilizing grade membranes, including the 4 dual‐layer sterile filters that already have a built‐in prefilter. This suggests that the improved filtration performance is a direct result of the change in properties of the NE caused by the Sterlitech 0.45 μm PVDF prefilter. The one exception was the highly asymmetric Millipore Express Plus, which had a capacity less than 80 g/m^2^ both with and without the prefilter. This low capacity is likely due to the very small pore size of this membrane (which has a bubble point of 5.72 ± 0.08 bar^23^ compared to 3.47 ± 0.07 bar for the Supor 0.2 μm sterile filter). The Sterlitech prefilter increased the capacity of the Durapore 0.2 μm membrane by a factor of five, and it increased the capacity of the Sartobran P by 18‐fold, going from a capacity of less than 50 g/m^2^ for the fresh NE to more than 940 g/m^2^ for the prefiltered NE. The highest capacity was obtained with the dual‐layer Sartopore 2 XLG (0.8/0.2 μm), with the NE prefiltered through the Sterlitech 0.45 μm PVDF prefilter showing a capacity of more than 4000 g/m^2^ during the sterile filtration.

### 3.5 Evaluation of other hydrophobic prefilters

**FIGURE 6:**
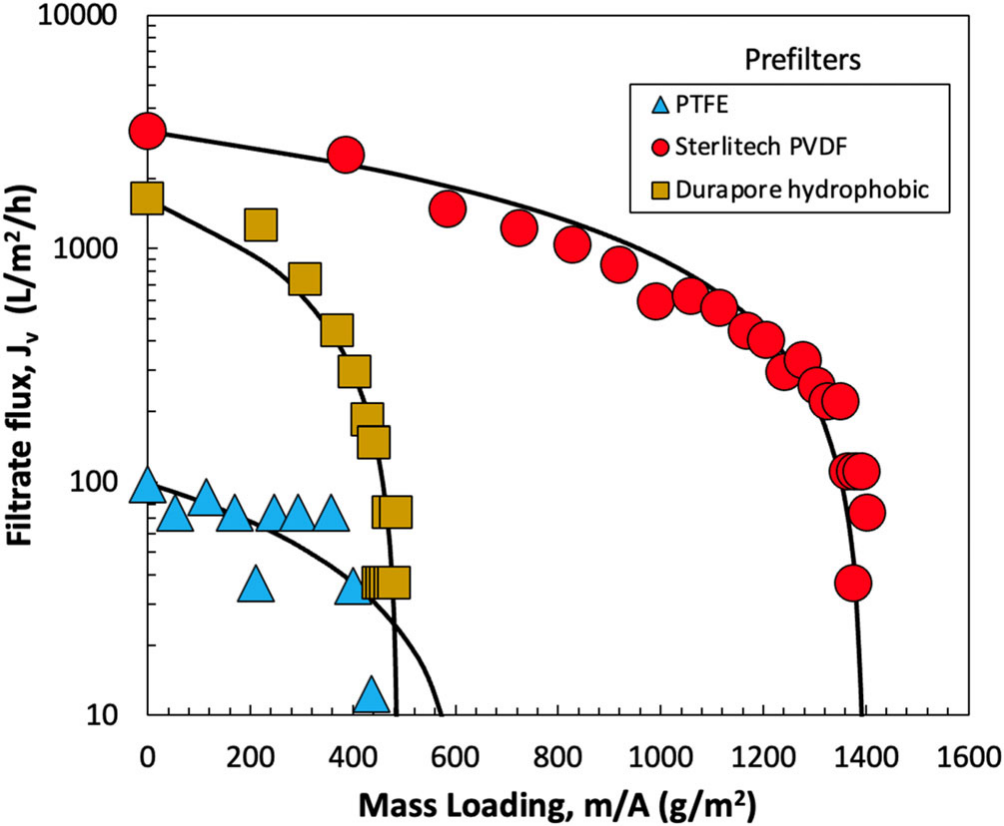
Filtrate flux as a function of mass loading during constant pressure filtration of the nanoemulsions through the Supor 0.2 μm sterile filter after passing through the PTFE, hydrophobic Durapore, and Sterlitech PVDF prefilters. Solid curves are model fits generated using the complete pore blockage model.

Based on the results with the Sterlitech 0.45 μm PVDF prefilter, we hypothesized that the hydrophobicity of the prefilter might play a critical role in altering the surface tension of the NE and in turn increasing the capacity of the sterile filter. To test this hypothesis further, experiments were conducted using two additional hydrophobic prefilters: a PTFE and a hydrophobic Durapore (both 0.45 μm rated). Both of these prefilters had contact angles of approximately 120°, similar to that of the Sterlitech 0.45 μm PVDF prefilter. Figure 6 presents the flux profiles during constant pressure filtration through the Supor 0.2 μm sterile filter after prefiltration through these three hydrophobic prefilters. The solid curves are again based on the complete pore blockage model (Equation 1). The initial flux through the Supor 0.2 μm sterile filtration membrane was greatest when using the Sterlitech 0.45 μm prefilter, with a value that was more than 20× greater than that after the PTFE prefilter. The choice of prefilter also had a significant impact on the sterile filter capacity, which was less than 600 g/m^2^ after prefiltration through the hydrophobic Durapore and PTFE prefilters (which is only slightly greater than the capacity without any prefilter) compared to a value of nearly 1400 g/m^2^ after prefiltration through the Sterlitech 0.45 μm PVDF prefilter. These large differences in performance were seen even though the three prefilters had very similar water contact angles.

**FIGURE 7:**
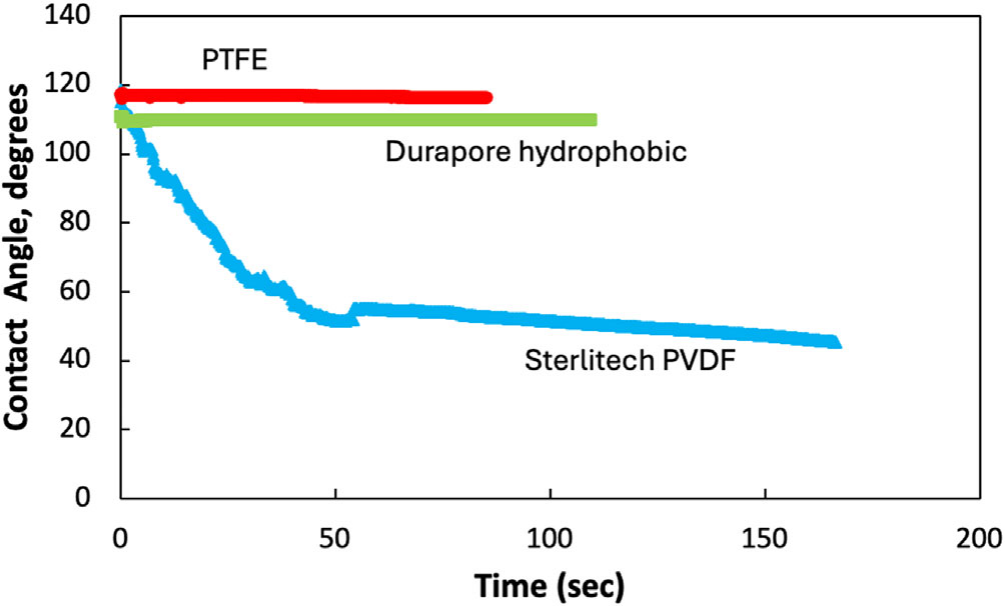
Dynamic water contact angles evaluated using the sessile drop method for the Sterlitech PVDF, hydrophobic Durapore, and PTFE prefilters as a function of time.

Additional insights into the surface properties of these prefilters were obtained by measuring the water contact angle as a function of time (Figure 7). All three prefilters exhibit similar initial water contact angles of approximately 120° as discussed previously. The contact angles for the PTFE and hydrophobic Durapore membranes remained nearly constant over the course of the experiment, decreasing by less than 5%. In contrast, the contact angle for the Sterlitech PVDF prefilter decreased significantly with time, stabilizing at a value around 50°, corresponding to a fairly hydrophilic material. This change in contact angle suggests a significant increase in the wettability of the pore structure, possibly due to some type of rearrangement in the accessibility of the more hydrophilic oxygen‐containing functionalities in the Sterlitech PVDF prefilter. This change in wettability may be directly related to the adsorptive characteristics, and in turn the highly effective performance, of the Sterlitech PVDF prefilter in this application.

## 4 DISCUSSION

Sterile filtration of nanoemulsions through 0.2 μm pore size sterilizing‐grade filters poses significant challenges due to membrane fouling, which leads to very low capacities and the need for large membrane areas. The data presented in this study show that it is possible to significantly increase the capacity of the sterile filter through the use of an appropriate prefilter. In particular, the capacity of the Supor 0.2 μm sterile filter could be increased by more than 2‐fold using either the Sterlitech 0.45 μm PVDF prefilter or the Supor 0.8 μm PES prefilter (from 650 g/m^2^ to more than 1300 g/m^2^). We also showed that the Sterlitech 0.45 μm prefilter was highly effective in enhancing the capacity of other sterile filters, in some cases providing more than a 10‐fold increase in capacity.

Interestingly, the prefiltration had relatively little effect on the size distribution of the nanodroplets within the NE, at least as determined by dynamic light scattering. Instead, the two best prefilters, the Supor 0.8 μm and Sterlitech 0.45 μm, both caused a significant reduction in the surface tension of the NE, likely due to the selective adsorption of more hydrophobic components from the NE (either free squalene or Span 85). Interestingly, the Sterlitech 0.45 μm prefilter also showed a highly time‐dependent contact angle, with the prefilter becoming much more hydrophilic as it is exposed to water. The importance of chemical interactions between the prefilter and specific components in the NE has not been previously identified as a critical factor determining the effectiveness of the prefilter in increasing the filterability of this class of drug product. Further studies will be required to more fully characterize these interactions, including the possible impact of the hydrophobic drug component on the NE and the nature of its interactions with the different prefilters. These efforts will hopefully lead to the development of novel prefilter chemistries specifically designed to enhance the sterile filtration of nanoemulsions for applications in drug delivery and as vaccine adjuvants.

## AUTHOR CONTRIBUTIONS

**Shreya Kapila:** Data curation, formal analysis, investigation, and writing–original draft. **Randal J. Soukup:** Funding acquisition, conceptualization, methodology, resources, and writing – review and editing. **Marissa E. Bradley:** Methodology, resources, and writing – review and editing. **David Boyd:** Methodology, resources, and writing – review and editing. **Andrew L. Zydney:** Conceptualization, funding, supervision, writing – review and editing.

## CONFLICT OF INTEREST STATEMENT

R.J.S., M.E.B., and D.B. are employees of Merck Sharp & Dohme LLC, a subsidiary of Merck & Co., Inc., Rahway, NJ, USA.

## Supporting information


**Figure S1:** Scanning electron micrograph images of the bottom and top surfaces of the Supor 0.8 μm polyethersulfone prefilter at several magnifications.
**Figure S2:** Scanning electron micrograph images of the bottom and top surfaces of the Sterlitech 0.45 μm PVDF prefilter at several magnifications.

## Data Availability

The data that support the findings of this study are available from the corresponding author upon reasonable request.
