# Supplementary material for: Optimizing sterile filtration of nanoemulsions through proper choice of prefilter properties
Source: Biotechnol Prog. 2025 Oct 31;42(1):e70087. doi: 10.1002/btpr.70087 (PMC12908093; doi:10.1002/btpr.70087)
Supplement: Supplementary file 1 — Figure S1: Scanning electron micrograph images of the bottom and top surfaces of the Supor 0.8 μm polyethersulfone prefilter at several magnifications. Figure S2: Scanning electron micrograph images of the bottom and top surfaces of the Sterlitech 0.45 μm PVDF prefilter at several magnifications. [file BTPR-42-e70087-s001.docx]

**Optimizing sterile filtration of nanoemulsions through proper choice of prefilter properties**

*Shreya Kapila, Randal J. Soukup, Marissa E. Bradley*, *David Boyd*, *and*

*Andrew L. Zydney*

**Supplementary Information**

Figures S1 and S2 show scanning electron micrographs of the top and bottom surfaces of the Supor 0.8 µm prefilter and the Sterlitech 0.45 µm prefilter. In both cases, the prefilters were flushed with water, removed from the filtration cell, and air dried in a fume hood at ambient temperature for 24 hours. The prefilters were then cut into small pieces, affixed to conductive pins with double-sided carbon tape, and sputter coated with a fine layer of gold/platinum using a Bal-tec SCD 050 Sputter coater. SEM images were obtained using a Zeiss SIGMA VP-FESEM at 3.0 kV.

**Figure S1:** Scanning electron micrograph images of the bottom and top surfaces of the Supor 0.8 µm polyethersulfone prefilter at several magnifications.

**Figure S2:** Scanning electron micrograph images of the bottom and top surfaces of the Sterlitech 0.45 µm PVDF prefilter at several magnifications.
